# Supplementary figures and images for: CagY-Dependent Regulation of Type IV Secretion in Helicobacter pylori Is Associated with Alterations in Integrin Binding
Source: mBio. 2018 May 15;9(3):e00717-18. doi: 10.1128/mBio.00717-18 (PMC5954226; doi:10.1128/mBio.00717-18)

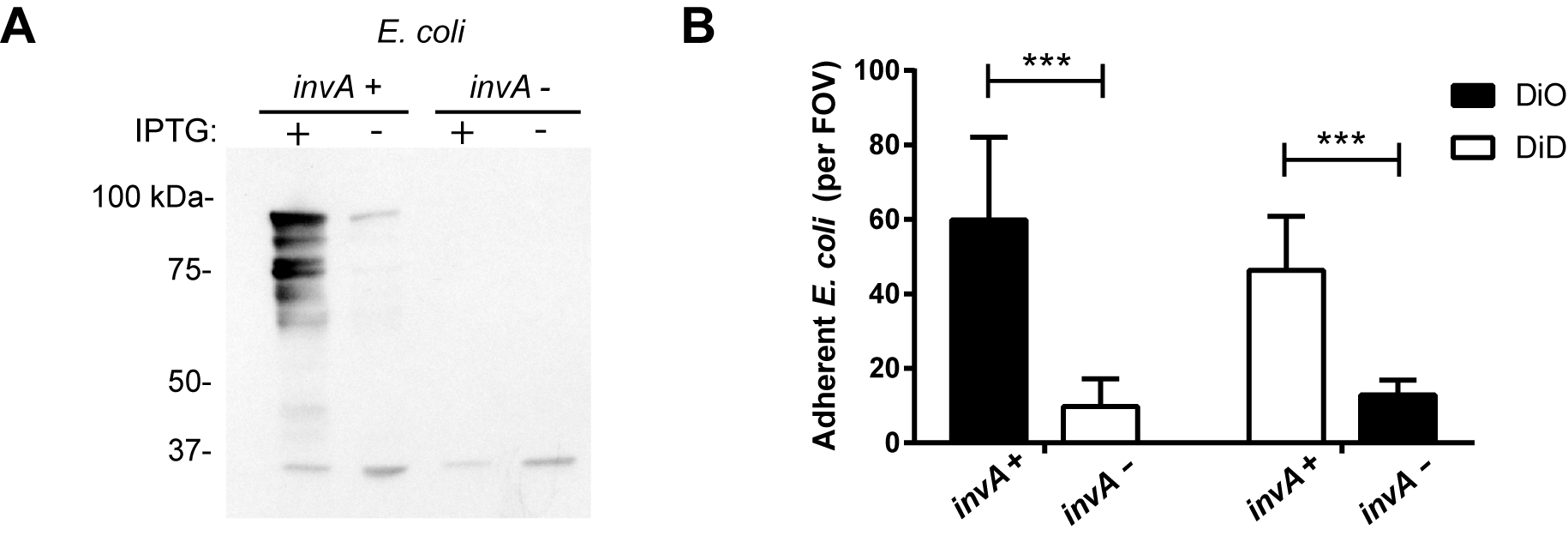

Supplement: FIG S1 [file mbo001183880sf1.tif]

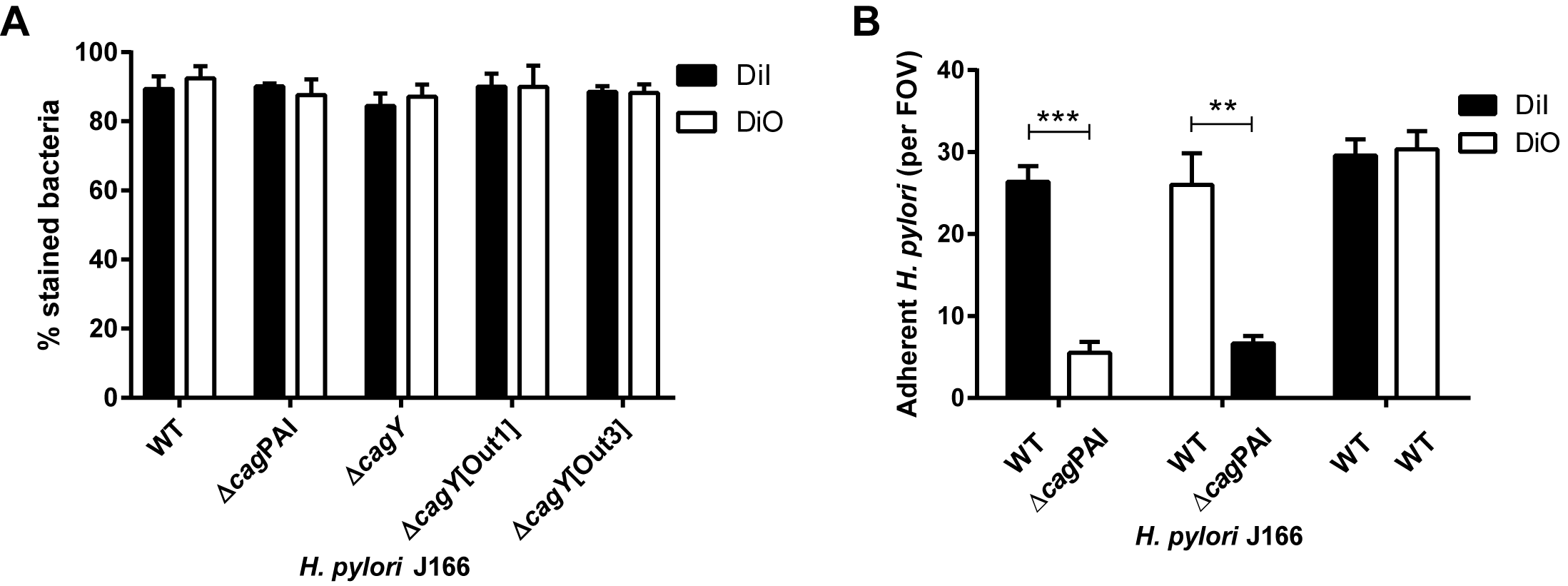

Supplement: FIG S2 [file mbo001183880sf2.tif]

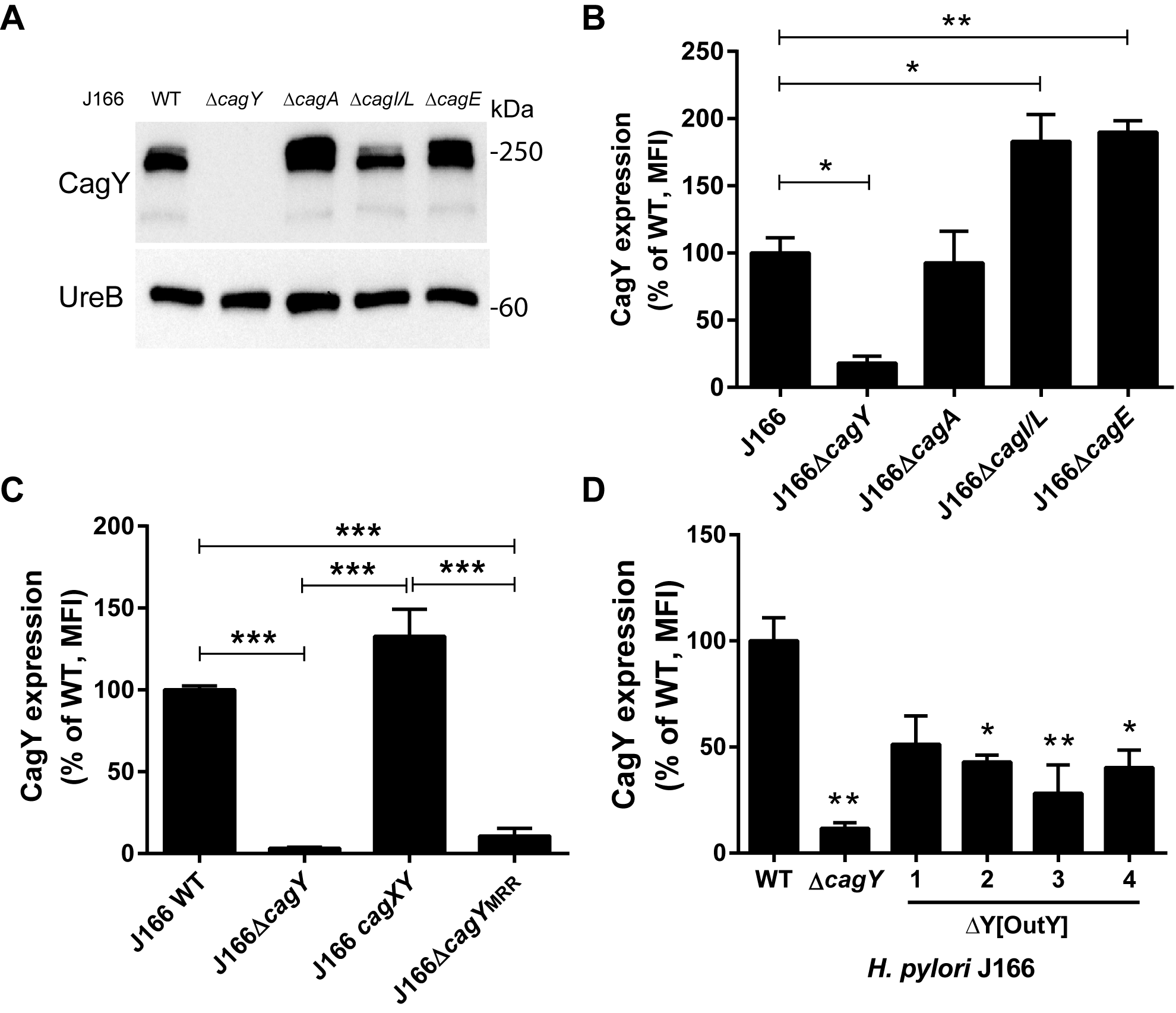

Supplement: FIG S3 [file mbo001183880sf3.tif]

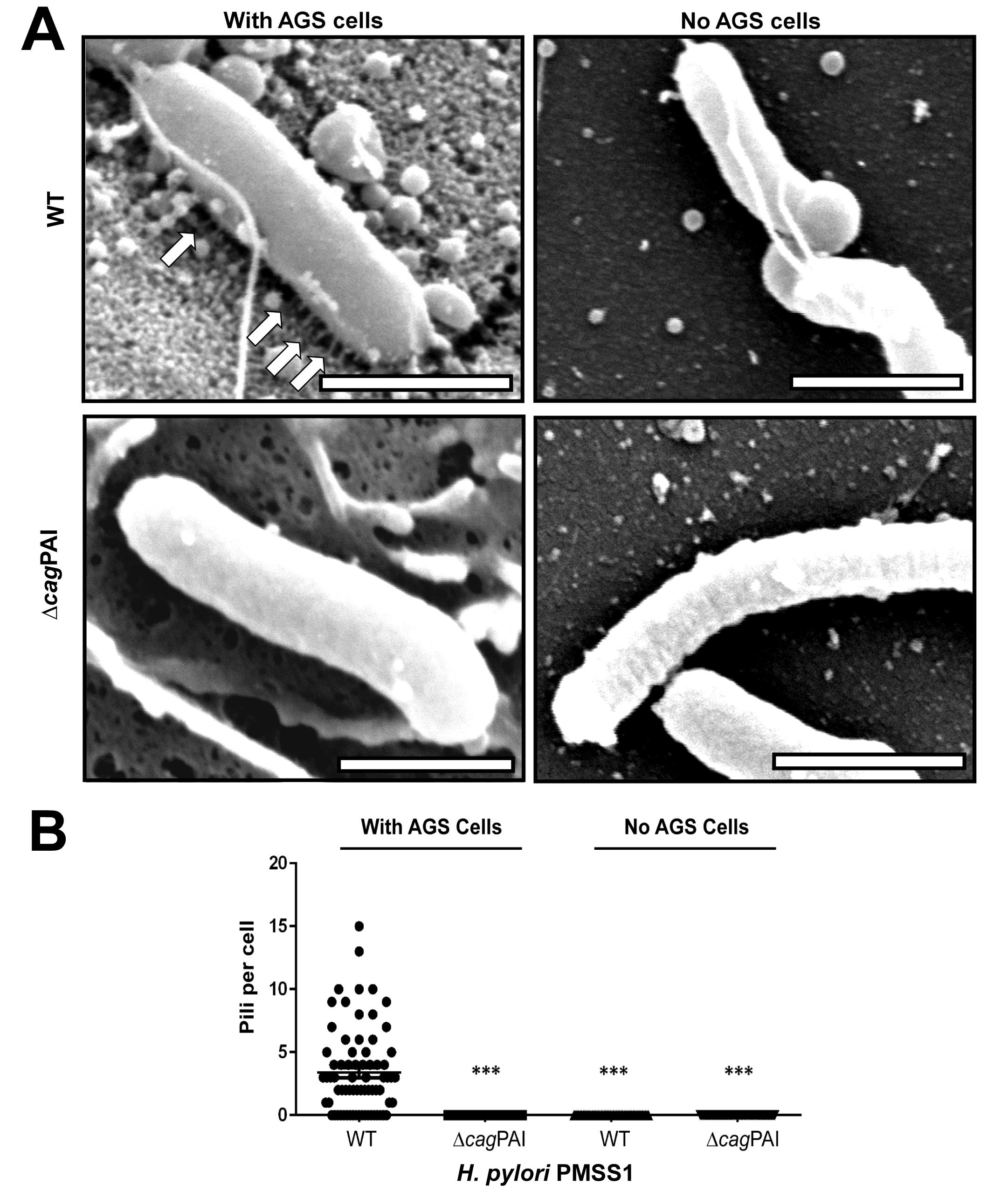

Supplement: FIG S4 [file mbo001183880sf4.tif]

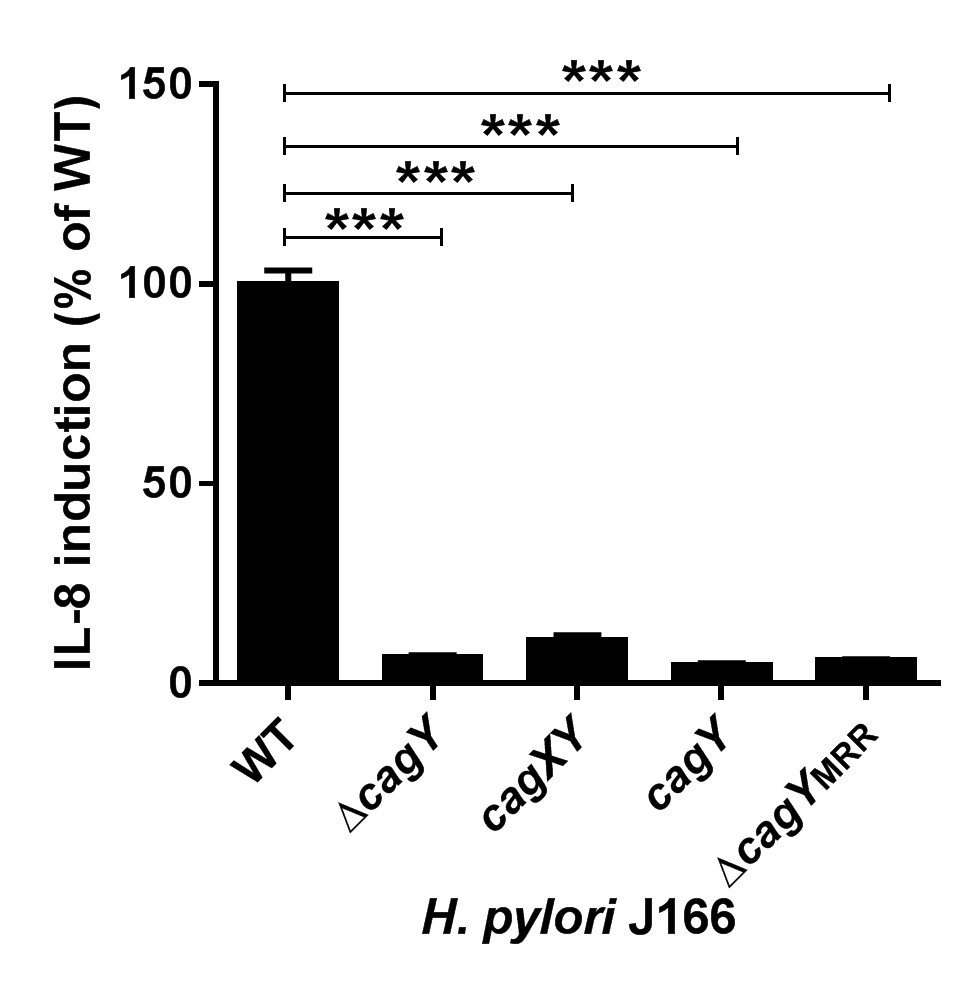

Supplement: FIG S5 [file mbo001183880sf5.tif]
