# Supplementary material for: CagY-Dependent Regulation of Type IV Secretion in Helicobacter pylori Is Associated with Alterations in Integrin Binding
Source: mBio. 2018 May 15;9(3):e00717-18. doi: 10.1128/mBio.00717-18 (PMC5954226; doi:10.1128/mBio.00717-18)
Supplement: TABLE S1 [file mbo001183880st1.docx]

| **Table S1. Primers used for PCR.** | |  |  |
| --- | --- | --- | --- |
| **Name** | **Sequence (5' to 3')** | |  |
| Deletion of *cagA* | |  |  |
| cagAup_XhoI | AAC CTC GAG GCT TTA CTT TAT GGT GAG CCA TAA C | |  |
| HP0547R_SacI | AAC GAG CTC GAT CAG GAT CAA ATG AAG CGA C | |  |
| HP0547F_HincII | AAC GTC GAC GAT TCG TTC AAG TTT TCC ACC | |  |
| HP0549F_NotI | AAC GCG GCC GCG AGT GCC TTA TGG CAC TAA AG | |  |
| Deletion of *cagE* | | | |
| cagE0F_XhoI | AAC CTC GAG GGA GCT TTA TTC TCA CTC TGA TCA GC | |  |
| HP0544:3L23_SacI | AAC GAG CTC CAG CTT GTT TGC TTG CCA CAA AC | |  |
| HP0544:2920U27_BamHI | AAC GGA TCC AGG CAA ATG TAT CAA CAA ATA GAG GAG | |  |
| HP0542:112L27_NotI | AAC GCG GCC GCC ACG CTA TTT AGT GCC ATA TCT TCA GG | |  |
| Deletion of *cagI+cagL* | | | |
| HP0541:206U24_XhoI | AAC CTC GAG CTG AAG CCG TAA GCA TGC CTA TGA | |  |
| HP0540:3L24_SacI | AAC GAG CTC CAC ATC TCT TTC CTC ACT TCA CGA | |  |
| HP0539:661U27_BamHI | AAC GGA TCC AGT AAG CGG CAA TAC CTA CAA GAA AGG | |  |
| HP0537:1003U27_NotI | AAC GCG GCC GCG TAG ATA GCG ATC CTA TGA TGA GCG AC | |  |
| Deletion of *cag1-6* | | | |
| HP0519:648L24_XhoI | AAC CTC GAG CTT GAA ATT TCA GCA ATG TCT TCC | |  |
| HP0520:134L21_SacI | AAC GAG CTC TAG CAC CGC TCA AGA CAC AAC | |  |
| HP0526:43L24_BamHI | AAC GGA TCC TCC CAT AAG AGA GCT GTT GCT ATC | |  |
| cagYJ166:5119U24_NotI | AAC GCG GCC GCG AGC CCG ACA TTA CCA AAC AAT AC | |  |
| cag1-6 stitchF | GTT GTG TCT TGA GCG GTG CTA TCC CAT AAG AGA GCT GTT GCT ATC | |  |
| cag1-6 stitchR | GAT AGC AAC AGC TCT CTT ATG GGA TAG CAC CGC TCA AGA CAC AAC | |  |
| Deletion of *cag9-25* | | | |
| HP0510:485U22_XhoI | AAC CTC GAG TGT ATG AAA CTT GCG CTA AGG C | |  |
| HP0546 up_SacI | AAC GAG CTC ATT CAG GAA CAA TAA ACC TAC TTG TCC | |  |
| HP0529:1348U24_BamHI | AAT GGA TCC GCA TTA TTG GCA TTC AAT TTG ACC | |  |
| HP0528:955L25_NotI | AAC GCG GCC GCG CTC CTC TTC AAT AAC GCT AGA GAC | |  |
| Deletion of *cag8*  HP0527:1041L25_XhoI | AAC CTC GAG CAC GAT AAG AAC AGC GAC TAC AAT G | | |
| cag8_7_SacI | AAC GAG CTC AAA GGC GTT AAG ACA TGA ATG AAG | | |
| cag8prom_HincII | AAC GTC GAC CAA CAA TTC CTC TTA AAA AAT ATT TGT AA | | |
| KanF_NotI | AAC GCG GCC GCG CTC GGA AGA GTA TGA AGA TGA A | | |
| cag8 stitchR | CAATTACAAATATTTTTTAAGAGGAATTGTTGATGAATGAAGAAAACGATAAACTTGAAA | | |
| cag8 stitchF | TTTCAAGTTTATCGTTTTCTTCATTCATCAACAATTCCTCTTAAAAAATATTTGTAATTG | | |
| Deletion of *cagY*_MRR_ | | | |
| HP0527:1349U27_XhoI | AAC CTC GAG AAG ACA AGT CAG AAG AAA TAA CTA ACG | |  |
| HP0527:2114L29_SacI | AAC GAG CTC CTG TTT CAA TCT TTC TAT AGC TTC TTT AG | |  |
| HP0527:4138U21_BamHI | AAC GGA TCC CAA GCC AAA ACT GAA GCT GAG | |  |
| HP0527:5101L23_Not | AAC GCG GCC GCG GCT CTA TGG AAG CAT AAA TAG G | |  |
| Stitch Left_forward | AAG ACA AGT CAG AAG AAA TAA CTA ACG | |  |
| Stitch Left_reverse | CGC TTT ATC TTT TTG TTG GAG TTC CTG TTT CAA TCT TTC TAT AGC TTC TTT AG | |  |
| Stitch Right_forward | CTA AAG AAG CTA TAG AAA GAT TGA AAC AGG AAC TCC AAC AAA AAG ATA AAG CG | |  |
| Stitch Right_reverse | GGC TCT ATG GAA GCA TAA ATA GG | |  |
| Amplification of antibiotic resistance cassettes | | | |
| rpsLF_SacI | AAC GAG CTC GAT GCT TTA TAA CTA TGG ATT AAA CAC | |  |
| C2-CatR_BamHI | AAC GGA TCC TTA TCA GTG CGA CAA ACT GGG AT | |  |
| C2-CatR_HincII | AAC GTC GAC TTA TCA GTG CGA CAA ACT GGG AT | |  |
| CAT-F_SacI | AAC GAG CTC GCG GAC AAC GAG TAA AAG AG | |  |
| CAT-R_BamHI | AAC GGA TCC GCA GGA CGC ACT ACT CTC G | |  |
| Kan-F_SacI | AAC GAG CTC GGT ACC CGG GTG AC | |  |
| Kan-R_BamHI | AAT GGA TCC TCT AGA GGA TCC CC | |  |
| Amplification of 3’ end of *cagY* for generation of antibody towards C-terminus (VirB10 homologue) | | | |
| cagYC-termBamHI | CGTGGATCCAGCAAACAAAATCAATTGAGT | |  |
| LJ39NotI | CGTAGCGGCCGCTTAATTGCCACCTTTGGGGCTTGTGGT | |  |
